# Downregulation of the endogenous opioid peptides in the dorsal striatum of human alcoholics

**DOI:** 10.3389/fncel.2015.00187

**Published:** 2015-05-12

**Authors:** Daniil Sarkisyan, Muhammad Z. Hussain, Hiroyuki Watanabe, Olga Kononenko, Igor Bazov, Xingwu Zhou, Olga Yamskova, Oleg Krishtal, Victor M. Karpyak, Tatiana Yakovleva, Georgy Bakalkin

**Affiliations:** ^1^Division of Biological Research on Drug Dependence, Department of Pharmaceutical Biosciences, Uppsala UniversityUppsala, Sweden; ^2^Government Degree CollegeMultan, Pakistan; ^3^Department for Cellular Membranology, Bogomoletz Institute of PhysiologyKyiv, Ukraine; ^4^Department of Functional Pharmacology, Institute for Neuroscience, Uppsala UniversityUppsala, Sweden; ^5^Department of Psychiatry and Psychology, Mayo ClinicRochester, MN, USA

**Keywords:** caudate nucleus, putamen, prodynorphin, proenkephalin, alcoholism

## Abstract

The endogenous opioid peptides dynorphins and enkephalins may be involved in brain-area specific synaptic adaptations relevant for different stages of an addiction cycle. We compared the levels of prodynorphin (*PDYN*) and proenkephalin (*PENK*) mRNAs (by qRT-PCR), and dynorphins and enkephalins (by radioimmunoassay) in the caudate nucleus and putamen between alcoholics and control subjects. We also evaluated whether *PDYN* promoter variant rs1997794 associated with alcoholism affects *PDYN* expression. Postmortem specimens obtained from 24 alcoholics and 26 controls were included in final statistical analysis. *PDYN* mRNA and Met-enkephalin-Arg-Phe, a marker of PENK were downregulated in the caudate of alcoholics, while *PDYN* mRNA and Leu-enkephalin-Arg, a marker of *PDYN* were decreased in the putamen of alcoholics carrying high risk rs1997794 C allele. Downregulation of opioid peptides in the dorsal striatum may contribute to development of alcoholism including changes in goal directed behavior and formation of a compulsive habit in alcoholics.

## Introduction

The endogenous opioid peptides dynorphins and enkephalins and their receptors have a critical role in drug and alcohol dependence (Shippenberg et al., 2007; Wee and Koob, 2010; Butelman et al., 2012). In clinics, the opioid antagonist naltrexone reduces alcohol drinking and relapse rates in subgroups of alcoholics (Volpicelli et al., 1992; Anton, 2008). In animal experiments, antagonists of the opioid receptors or genetic deletion of these receptors alter alcohol consumption (Shippenberg et al., 2007; Walker and Koob, 2008; Wee and Koob, 2010). Animal studies propose that drug/alcohol induced changes in prodynorphin (*PDYN*) and proenkephalin (*PENK*) expression underlie neuroplastic adaptations critical for addiction (Shippenberg et al., 2007; Walker and Koob, 2008; Wee and Koob, 2010; Butelman et al., 2012). These changes may be brain-area specific, and may differentially contribute to specific stages of an addiction cycle (Yuferov et al., 2009; Butelman et al., 2012; Bazov et al., 2013).

Development of drug/alcohol dependence may be viewed as a maladaptive habit formation during transition from recreational to compulsive use that is associated with a diminishing cognitive control over drug seeking and taking behavior (Everitt and Robbins, 2005; Shippenberg et al., 2007; Wee and Koob, 2010; Robison and Nestler, 2011; Butelman et al., 2012). These processes are characterized by a shift from prefrontal cortical to striatal control over drug/alcohol use, and a progression from the ventral to dorsal striatum in the addicted brain (Everitt and Robbins, 2005; Vollstadt-Klein et al., 2010). Molecular adaptations developed over the course of drug and alcohol exposure in the dorsal striatum may be critical for the formation of a compulsive habit (Belin et al., 2009).

Striatal subregions may be differentially involved in addiction cycle (Everitt and Robbins, 2005; Balleine and O'Doherty, 2010; Vollstadt-Klein et al., 2010; Butelman et al., 2012). The caudate nucleus participates in control of goal-directed actions, and thus may influence goal-directed alcohol seeking. The putamen has key roles in habit formation, and may participate in the development of habitual alcohol use. Animal research demonstrated that the drugs- or alcohol-induced changes in *PDYN* and *PENK* expression in the dorsal striatum may contribute to the development of addictive state (Spangler et al., 1993; Shippenberg et al., 2007; Walker and Koob, 2008; Wee and Koob, 2010; Butelman et al., 2012).

The aim of the present study was to examine whether *PDYN* and *PENK* and their peptide products are involved in adaptive processes in the dorsal striatum in human alcohol dependent individuals. We compared the levels of *PDYN* and *PENK* mRNA and four opioid peptides in post-mortem human specimens from human alcoholics and control subjects. Dynorphin A (Dyn A), dynorphin B (Dyn B) and Leu-enkephalin-Arg (LER) derived from PDYN, and Met-enkephalin-Arg-Phe (MEAP) derived from PENK were analyzed. PENK is processed to Met-enkephalin and Leu-enkephalin in a 4:1 ratio, as well as MEAP and Met-enkephalin-Arg-Gly-Leu (Akil et al., 1984; Christensson-Nylander et al., 1985; Evans et al., 1985; Nyberg et al., 1986; Nylander et al., 1994; Yakovleva et al., 2006; Slominski et al., 2011) (http://www.uniprot.org/uniprot/P01210). The opioid peptide sequences in PDYN are all Leu-enkephalin with C-terminal extension with an arginine residue. This sequence is also unique to this prohormone (Christensson-Nylander et al., 1985; Nyberg et al., 1986; Nylander et al., 1994) (http://www.uniprot.org/uniprot/P01213) while Leu-enkephalin is extended from the C-terminus with Lys in PENK. In previous studies MEAP is measured as a marker of PENK, while Dyn A, Dyn B and LER as markers of the PDYN system (Christensson-Nylander et al., 1985; Evans et al., 1985; Nyberg et al., 1986; Nylander et al., 1994; Yakovleva et al., 2006; Slominski et al., 2011).

Dyn A and Dyn B function as ligands for κ-opioid receptor (KOR), whereas LER is potent agonist for δ- (DOR) and μ- (MOR) opioid receptors and MEAP may activate κ- and μ-opioid receptors (Christensson-Nylander et al., 1985; Iyengar et al., 1987; Kamei et al., 1994; Mansour et al., 1995; Benyhe et al., 1997; Nylander et al., 1997). Single nucleotide polymorphism (SNP; rs1997794) in *PDYN* promoter was found to be associated with alcohol and cocaine dependence (Xuei et al., 2006; Yuferov et al., 2009) and heroin addiction (Clarke et al., 2009). The C, high risk allele of this SNP destroys binding element for AP-1 transcription factor and may affect *PDYN* transcript abundance in human brain (Babbitt et al., 2010; Taqi et al., 2011). Therefore, we here evaluated whether this SNP impacts changes in striatal *PDYN* expression associated with alcoholism.

## Materials and methods

### Human samples/case selection

Tissues were collected at the New South Wales Tissue Resource Centre (NSW TRC), University of Sydney, Australia (http://www.braindonors.org; http://rp-host.www.pathology.med.usyd.edu.au/trc/index.php) (Sheedy et al., 2008). Analysis was initiated with 30 controls and 30 chronic alcoholics with known smoking history (Tables 1, 2). All subjects were males of European descent. Alcohol dependent subjects fulfilled criteria for Diagnostic and Statistical Manual for Mental Disorders, 4th edition (DSM-IV) and National Health and Medical Research Council/World Health Organization Criteria, and consumed greater than 80 g of ethanol per day for the majority of their adult lives (Harper et al., 1988). Controls either did not drink alcohol at all or were social drinkers who consumed less than 20 g of ethanol per day on average. Control cases were matched to alcoholic cases by sex, race, age, brain pH and post-mortem interval (PMI). Cases with a history of polydrug abuse (with evidence that the individual abused other drugs such as cocaine or heroin) or with medical complications such as Wernicke-Korsakoff syndrome or alcoholic cases with concomitant diseases were excluded. Cases with a prolonged agonal life support or cases with a history of cerebral infarction, head injury, or neurodegenerative diseases (e.g., Alzheimer's disease) were also excluded. Samples were handled by qualified pathologists under ethical clearance from Sydney South West Area Health Service, Human Ethics Committee (X03-0074). Informed written consent was obtained from the next of kin. The study was approved by the Swedish Central Ethical Review Board.

**Table 1:** **Demographic data and tissue characteristics of control subjects**.

**Subject no**	**Age (years)**	**PMI (hours)**	**Brain pH**	**Storage time (months)**	**RQI**	**Smoking history**
1^*^	54	29	6.8	68	7.3	No
2^*^	60	13	6.59	168	8.9	NA
3^*^	53	27	6.64	48	7.7	NA
4^*^	43	66	6.2	82	6	NA
5^*^	82	23.5	6.4	92	5.9	NA
6^*^	68	45.5	6.12	23	6	No
7^*^	61	27.5	6.25	29	5.5	No
8	50	19	6.26	102	7.8	Yes
9^*^	57	18	6.6	79	7.8	Yes
10^*^	54	28	6.38	12	8.3	Yes
11^*^	53	16	6.5	9	8.9	Yes
12	63	72	6.9	82	8.1	Yes
13^*^	56	37	6.76	84	8.2	Yes
14^*^	73	51	6.82	49	4	Yes
15	60	25	6.7	74	6	No
16^*^	36	34	6.67	13	5.9	Yes
17^*^	58	28	5.92	11	5.7	Yes
18^*^	69	52	6.95	9	8.6	No
19^*^	37	14.5	6.46	9	7.7	No
20	59	40	6.53	22	6.4	Yes
21^*^	50	30	6.37	22	7.2	Yes
22^*^	69	16	6.6	80	8.2	Yes
23^*^	46	25	6.65	119	8.5	NA
24^*^	60	21.5	6.66	41	7.8	No
25^*^	73	38.5	6.28	19	8.1	Yes
26^*^	55	20	6.5	174	8.6	Yes
27^*^	59	43	6.69	34	8.4	Yes
28^*^	64	9.5	6.94	49	8.3	Yes
29^*^	47	38	6.74	24	8.6	Yes
30^*^	63	24	6.94	52	7.3	Yes
Mean ±	57.7 ± 9.2	31.1 ± 14.8	6.56 ± 0.25	56.0 ± 43.7	7.4 ± 1.2	
SD						
Mean^*^ ±	57.7 ± 10.7	29.8 ± 13.3	6.6 ± 0.26	53.8 ± 45.2	7.4 ± 1.3	
SD^*^						

* *Subjects included in the final statistical analysis*.

**Table 2:** **Demographic data and tissue characteristics of alcohol dependent subjects**.

**Subject no**	**Age (years)**	**PMI (hours)**	**Brain pH**	**Storage time (months)**	**RQI**	**Smoking history**
1^*^	43	29	6.29	56	6.2	Yes
2^*^	70	32	6.05	143	6.7	NA
3	37	17	6.33	115	6.6	No
4	81	36	6.44	103	8.7	Yes
5^*^	50	24	6.59	130	8.4	Yes
6^*^	57	43	6.46	48	8.6	Yes
7^*^	56	65	6.47	17	5.9	Yes
8^*^	58	20	6.64	59	6.8	Yes
9^*^	60	16.5	6.48	46	8.6	Yes
10^*^	60	51	6.7	66	7.3	No
11^*^	61	27.5	5.87	19	5.3	Yes
12^*^	55	48	7.02	25	8.6	Yes
13	41	54	6.7	86	8.2	Yes
14^*^	53	60	6.75	86	7.6	Yes
15^*^	63	25.5	6.21	31	5	Yes
16^*^	73	43.5	6.59	39	7	No
17^*^	55	17	6.85	22	8.5	No
18^*^	69	22	5.82	11	5.7	Yes
19	70	62	6.82	92	7.1	Yes
20	65	72	6.88	14	7.1	Yes
21^*^	58	44.5	6.47	15	8.2	Yes
22^*^	50	17	6.3	100	6.4	NA
23^*^	56	15	6.66	113	7.8	NA
24^*^	59	24	6.57	116	6.6	No
25^*^	52	45.5	6.78	90	5.8	Yes
26^*^	70	33.5	6.24	120	3.3	Yes
27	54	17	6.41	142	8.3	Yes
28^*^	56	22	6.52	116	8.3	Yes
29^*^	42	41	6.5	82	7.7	No
30^*^	64	39	6.76	23	9	Yes
Mean ±	57.9 ± 9.8	35.5 ± 16.2	6.5 ± 0.28	70.8 ± 42.3	7.2 ± 1.3	
SD						
Mean^*^ ±	57.9 ± 7.7	33.6 ± 13.9	6.5 ± 0.29	65.5 ± 41.4	7.1 ± 1.4	
SD^*^						

* *Subjects included in the final statistical analysis*.

### Gene expression analysis by qRT-PCR

#### Total RNA isolation and cDNA synthesis

Total RNA was purified with RNeasy Lipid Tissue Mini kit (QIAGEN, Maryland, USA) using TRIzol Reagent (QIAGEN, Maryland, USA) and treated with RNase-free DNase I on-column. RNA Quality Indicator (RQI) was measured using Bio-Rad Experion system (Bio-Rad Laboratories, Hercules, CA) with Eukaryote Total RNA StdSens assay according to the manufacturer's protocol. RNA samples with RQI values above 5.0 are generally considered suitable for quantitative real-time polymerase chain reaction (qRT-PCR) (Fleige and Pfaffl, 2006; Fleige et al., 2006). Average values of RQI in controls and alcoholics were 7.39 ± 1.27 and 7.23 ± 1.41 respectively, demonstrating high quality of isolated RNA. Reverse transcription of total RNA was performed with cDNA iScript kit (Bio-Rad Laboratories, Hercules, CA, USA) using CFX96™ Real-Time Detection System (Bio-Rad Laboratories, Hercules, CA, USA) according to the manufacturer's protocol.

#### Quantitative real-time PCR (qRT-PCR)

qRT-PCR was performed on CFX96TM Real-Time Detection System (Bio-Rad Laboratories, Hercules, CA). For SYBR Green-based assays, the reaction mixture consisted of cDNA, 5 × HOT FIREPol® EvaGreen® qPCR Mix Plus (Solis BioDyne, Tartu, Estonia) and forward and reverse primers (Supplementary Table S2). The following conditions were applied for the three-step qRT-PCR reaction; 95°C for 15 min followed by 40 cycles of amplification at 95°C for 15 s, annealing temperature for 61.6°C for 20 s and elongation at 70°C for 20 s. Melting curves were analyzed to ensure primer specificity and lack of primer dimers. To ensure correct amplification, PCR products were separated on agarose gel and sequenced in both directions. Samples were analyzed in triplicates. Using the previously developed approach for analysis of reference genes (Johansson et al., 2007), the beta-actin (ACTB), TATA box binding protein (TBP) and beta-2 microglobulin (B2M) for putamen; and the beta-actin (ACTB), TATA box binding protein (TBP) and glyceraldehyde-3-phosphate dehydrogenase (GAPD) for caudate nucleus were used for normalization. No fluorescence was observed in samples not containing template cDNA (no template control) or in negative controls prepared by the omission of reverse transcriptase.

#### DNA purification and genotyping

DNA was purified from human brain samples using Wizard Genomic DNA Purification kit (Promega, Madison, USA). Genotyping of SNP rs1997794 located in *PDYN* promoter was performed by allelic discrimination using TaqMan SNP Genotyping Assay C_11670951_10 (Applied Biosystems, Foster City, CA, USA) according to the manufacturer's protocol. Polymerase chain reactions were set up in a total volume of 10 μl, including 1 × iTaq Universal Probes Supermix (Bio-Rad, Sunnyvale, CA, USA), 1 × TaqMan SNP Genotyping Assay (Applied Biosystems), and 10 ng of template DNA using the BioRad C1000 Thermal Cycler (CFX96 Real-Time System) (Bio-Rad). After an initial denaturation step for 10 min at 95°C, each cycle consisted of denaturation for 15 s at 95°C and annealing and primer extension for 60 s at 62°C for a total 40 cycles. The rs1997794 variant pattern was set up with DNA from positive controls previously genotyped (Taqi et al., 2011).

#### Radioimmunoassay (RIA)

The procedure has been described elsewhere (Christensson-Nylander et al., 1985; Merg et al., 2006). Briefly, 1 M hot acetic acid was added to finely powdered frozen brain tissues, and samples were boiled for 5 min, ultrasonicated and centrifuged. Tissue extracts were run through SP-Sephadex ion exchange C-25 column, and peptides were eluted and analyzed by RIA. Anti-Dyn A antibody demonstrated 100% molar cross-reactivity with Dyn A (9–17) and <0.1% molar cross-reactivity with Dyn B, Dyn A (1–8), α-neoendorphin, Leu-enkephalin, and big dynorphin. Anti-Dyn B antiserum showed 100% molar cross-reactivity with big dynorphin, 0.8% molar cross-reactivity with Leu-morphine (29 amino acid C-terminally extended Dyn B), and <0.1% molar crossreactivity with Dyn A (1–17), Dyn A (1–8), α-neoendorphin, and Leu-enkephalin (Yakovleva et al., 2006). Cross-reactivity of LER antiserum with Dyn A, Dyn B and Leu- and Met-enkephalin was <0.1% molar, with α-neoendorphin 0.5% molar, with Dyn A (1–8) 0.7% molar, with MEAP 1% molar and with Met-enkephalin-Arg 10% molar. Cross-reactivity of MEAP antiserum with Met-enkephalin, Met-enkephalin-Arg, Met-enkephalin-Arg-Gly-Leu, Leu-enkephalin and LER was <0.1% molar (Nylander et al., 1997).

Dyn A, Dyn B, and LER RIA readily detected these peptides in the striatum, hippocampus, and frontal cerebral cortex of wild-type mice (Nguyen et al., 2005) but not in Pdyn knockout mice (for details, see Merg et al., 2006); thus the assay was highly specific and not sensitive to the presence of contaminants in brain tissues. Protein concentrations were estimated by DC protein assay (Bio-Rad, Laboratories, Hercules, CA, USA). The peptide content was calculated with the GraphPad Prism (GraphPad Software Inc., San Diego, CA, USA) and presented in fmol/mg of tissue.

#### Statistical analyses

Statistical analysis was carried out using the R statistical software (http://www.R-project.org/) libraries car and lsmeans. The assumption of conducting analyses of variance (ANOVAs and ANCOVAs) were verified: (i) residuals being normally distributed according to Kolmogorov-Smirnov test, and (ii) error variance being homoscedastic across groups according to Levene test. Dependent variables (DVs) that failed assumptions (i) or (ii) were analyzed by Kruskal-Wallis one-way non-parametric ANOVA. Analyses were conducted in four general steps. First, data was subjected to one-way ANOVAs with group as between factor for each of 12 DVs (6 molecular levels including *PDYN* and *PENK* mRNA, Dyn A, Dyn B, LER, and MEAP, in 2 brain regions). Second, we have used backward stepwise regression to refine the model by determining the relative importance of each predictor and its statistical significance. The predictors were selected among demographic parameters and tissue characteristics group (two levels: controls and alcoholics), genotype for PDYN system only (two levels: the CC and CT genotypes vs. the TT genotype of *PDYN* rs1997794), smoking (two levels: smokers and non-smokers), age, brain pH, PMI, and RQI (for mRNA DVs only). Because, an equation containing an excessive number of independent variables may be overfitted, we applied a rule of thumb (six cases per variable) to determine the number of independent variables in the analysis. Library Hmisc and Bonferroni correction for multiple tests were used to compute significance of Spearman correlations for demographic parameters (age, smoking) and tissue characteristics (PMI, pH, RQI) to ensure that the regression's predictors were not collinear. Original models for backward stepwise regressions always controlled for demographic parameters and tissue characteristics. When final models showed significant main effect of smoking or significant group × smoking interaction only subjects with known smoking history were analyzed. According to the general approach developed for identification of overly influential points (Faraway, 2002) we identified 10 subjects (4 controls and 6 alcoholics) with Cook's distances >1, and each of these ten was identified as such in two or more regressions. We excluded these subjects from further analysis because of high likelihood of them belonging to different subpopulations, or of their tissue characteristics being heavily influenced by post-mortem procedures. This resulted in Cook's distances <0.42 for the linear models involving remaining 50 subjects. Third, analyses of covariance (One-Way ANCOVA) with group as between factor were performed. Fourth, data was subjected to Two-Way ANCOVAs with group and genotype as between factors. ANCOVAs were followed by *post-hoc* pairwise two-way Student's *t*-tests on least squares means (between group and genotype factors, controlled for confounds) with Sidak correction for multiple comparisons. A *p*-value of 0.05 after multiple testing corrections was accepted as statistically significant.

## Results

Analyzing demographic and clinical data and tissue characteristics *t*-test showed no significant differences in age [*t*_(45)_ = 0.084, *p* = 0.93], post-mortem interval (PMI) [*t*_(47)_ = 0.95, *p* = 0.35], brain pH values [*t*_(46)_ = −0.91, *p* = 0.37], RNA quality indicator (RQI) [*t*_(46)_ = −0.99, *p* = 0.32] and Firsher's exact test showed no significant difference in number of smokers (odd ratio = 0.78, *p* = 1) between alcoholics and controls.

To examine whether the endogenous opioid peptide system undergoes adaptive changes in the striatum of human alcoholics we analyzed the levels of *PDYN* and *PENK* mRNA, PDYN-derived Dyn A, Dyn B and LER, and PENK-derived MEAP in post-mortem samples of the caudate and putamen of alcoholics (24 subjects) and controls (26 subjects).

**Figure 1:**
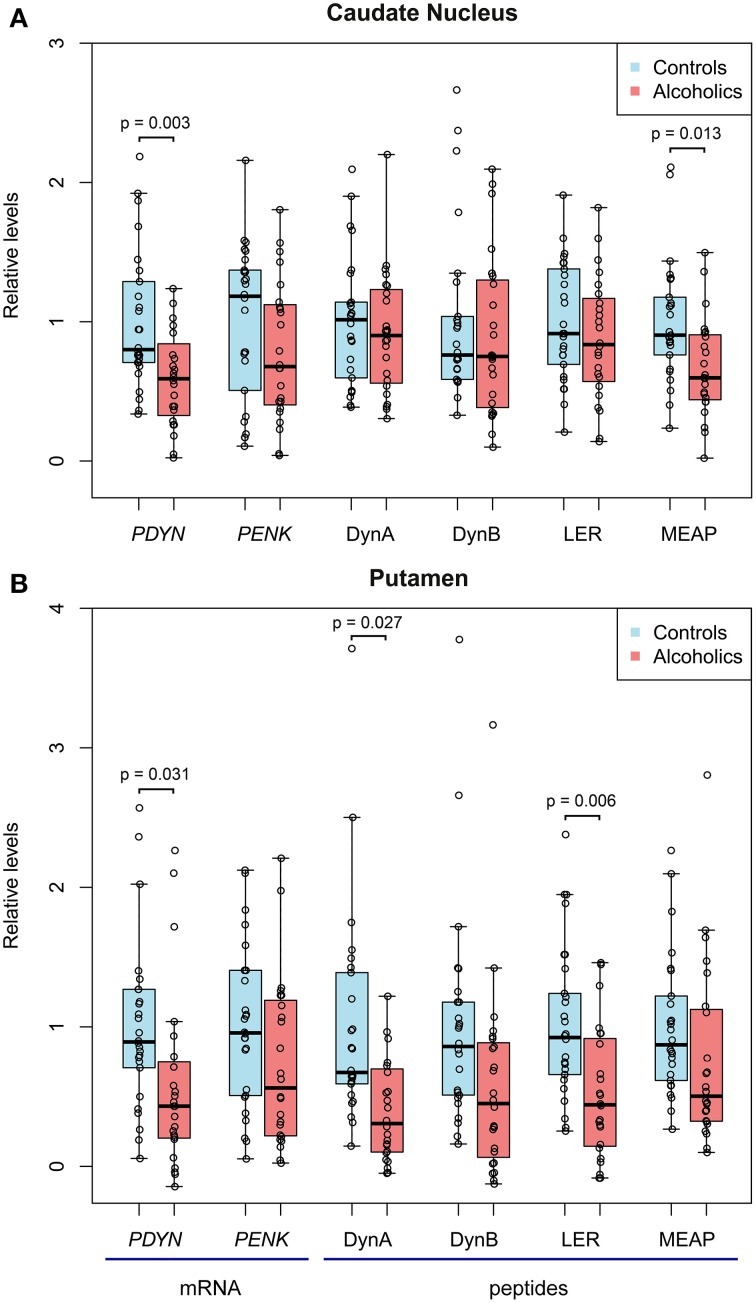
**The relative levels of opioid *PDYN* and *PENK* mRNA, PDYN derived Dyn A, Dyn B and Leu-enkephalin-Arg (LER) opioid peptides, and PENK-derived Met-enkephalin-Arg-Phe (MEAP) opioid peptide in the caudate nucleus (A) and putamen (B) of alcoholics and controls**. mRNA and peptide levels were normalized to geometric mean of reference genes and mg of tissue, respectively, and individual data points were corrected for demographic and tissue co-factors. Data are shown as median, upper and lower quartiles with the mean value in controls taken as a unit. In controls, *PDYN* and *PENK* mRNA levels before scaling were 0.379 and 0.799 relative units in caudate nucleus, and 0.677 and 0.947 relative units in putamen, respectively. In controls, Dyn A, Dyn B, LER, and MEAP before scaling were 396.4, 20.6, 4.5, and 124.8 fmol/mg of tissue in caudate nucleus; and 634.2, 20.0, 7.9, and 53.9 fmol/mg of tissue in putamen, respectively.

For the caudate nucleus, One-Way ANCOVAs revealed effect of alcoholism on *PDYN* mRNA [*F*_(1, 36)_ = 7.6, *p* = 0.009] and MEAP [*F*_(1, 40)_ = 6.85, *p* = 0.013]. *Post-hoc t*-test showed downregulation of *PDYN* (1.7-fold; *p* = 0.003) and MEAP (1.5-fold; *p* = 0.013) in alcoholics (Figure 1A). PMI [*F*_(1, 36)_ = 6.7, *p* = 0.013] and smoking [*F*_(1, 36)_ = 4.6, *p* = 0.039] effects were revealed for *PDYN*.

For the putamen, One-Way ANCOVAs revealed effect of alcoholism on *PDYN* mRNA [*F*_(1, 35)_ = 7.3, *p* = 0.011], Dyn A [*F*_(1, 39)_ = 5.3, *p* = 0.027] and LER [*F*_(1,40)_ = 10.3, *p* = 0.003]. *Post-hoc t*-test showed downregulation of *PDYN* (1.7-fold; *p* = 0.051), Dyn A (1.8-fold; *p* = 0.027) and LER (1.9-fold; *p* = 0.006) in alcoholics (Figure 1B). A pH effect was revealed for *PDYN* [*F*_(1, 35)_ = 5.6, *p* = 0.024]. Group × smoking interactions were revealed for *PDYN* [*F*_(1, 35)_ = 5.6, *p* = 0.024] and LER [*F*_(1, 35)_ = 6.5, *p* = 0.015].

*PDYN* promoter SNP (rs1997794) associated with alcoholism (Xuei et al., 2006; Yuferov et al., 2009) may form non-canonical AP-1 binding site and influence gene expression in human brain (Taqi et al., 2011). We next examined whether adaptive *PDYN* responses to alcohol are modulated by this SNP. The Fisher's exact test revealed no significant differences in distribution of the promoter SNP rs1997794 genotypes (*p* = 0.24) and a trend in that of alleles (*p* = 0.10) between alcoholics and control subjects (Supplementary Table S1).

**Figure 2:**
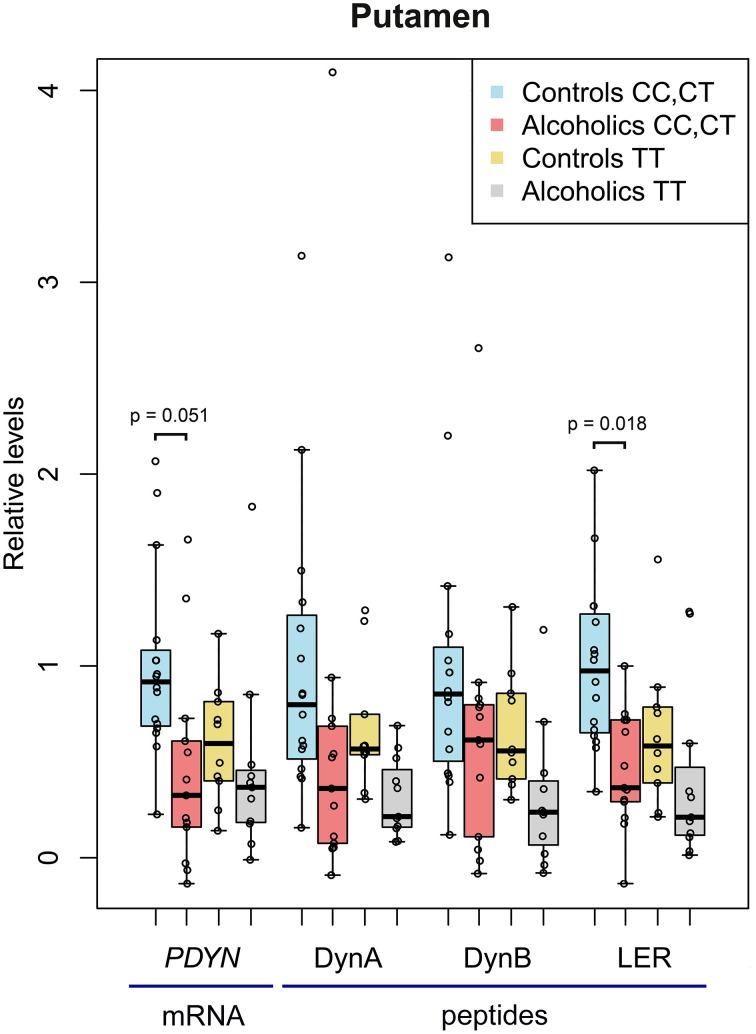
**The relative levels of *PDYN* mRNA and PDYN derived opioid peptides in the putamen of alcoholic and control subjects carrying the C, high risk allele (CC and CT genotypes) and the T allele (TT genotype) of *PDYN* promoter SNP rs1997794**. For details, see Figure 1 legend. In controls carrying the CC+CT genotype, *PDYN* mRNA, and Dyn A, Dyn B, and LER levels before scaling were 0.847 relative units, and 758.5, 24.0, and 9.6 fmol/mg of tissue, respectively.

Two-Way ANCOVAs with group (controls vs. alcoholics) and *PDYN* genotype (CC and CT genotypes vs. TT genotype; subjects with the C, high risk genotype were pooled) as between factors revealed no additional significant effects in the caudate nucleus, while uncovered a significant main effects of alcoholism for *PDYN* [*F*_(1, 35)_ = 7.3, *p* = 0.011], Dyn A [*F*_(1, 39)_ = 5.3, *p* = 0.027] and LER [*F*_(1, 35)_ = 10.3, *p* = 0.003], and a trend for Dyn B [*F*_(1, 36)_ = 3.84, *p* = 0.058] in the putamen (Figure 2). A genotype effect was revealed for Dyn A [*F*_(1, 39)_= 4.3, *p* = 0.044] and Dyn B [*F*_(1, 36)_ = 4.2, *p* = 0.049], but no biologically meaningful *post-hoc* differences were significant. A pH effect was revealed for *PDYN* [*F*_(1, 35)_ = 5.6, *p* = 0.024]. A group × smoking interaction was revealed for *PDYN* [*F*_(1, 35)_ = 5.6, *p* = 0.024] and LER [*F*_(1, 35)_ = 6.5, *p* = 0.015]. For the combined CC and CT genotypes *post-hoc t*-test showed downregulation of *PDYN* (2.2-fold; *p* = 0.051) and LER (2.2-fold; *p* = 0.018) in alcoholics.

Thus, *PDYN* mRNA and MEAP were downregulated in the caudate nucleus, while *PDYN* mRNA, Dyn A and LER were downregulated in the putamen of alcoholics. In the putamen, the *PDYN* and LER downregulation was significant in the subgroup of subjects carrying C, high risk allele of *PDYN* SNP rs1997794.

## Discussion

Our previous analysis identified upregulation of dynorphins and κ-opioid receptor in the dorsolateral prefrontal cortex, orbitofrontal cortex and hippocampus, the cognitive brain regions involved in control of impulsivity, decision-making, and learning and memory in human alcoholics (Taqi et al., 2011; Bazov et al., 2013). In contrast, downregulation of *PDYN* mRNA and *PDYN* and *PENK* derived peptides was demonstrated in the putamen and caudate nucleus in human alcoholics in the present study. The brain area specific up- and down-regulation of *PDYN* expression may be relevant for different aspects of alcohol dependence, specifically in the cognitive areas for impairment of cognitive control of addictive behavior, and in the dorsal striatum for changes of goal directed behavior and formation of a compulsive habit, respectively.

Study of primates chronically exposed to alcohol demonstrated increased spine density, enhanced glutamatergic transmission and increased intrinsic excitability in the putamen (Cuzon Carlson et al., 2011). The balance of inhibitory/excitatory transmission was proposed to be shifted toward a persistent increase in synaptic activation of putamen output as a consequence of prolonged heavy drinking and relapse. Downregulation of the dynorphin/κ-opioid receptor system, which activation is generally characterized by inhibitory influences on glutamate release and neuronal excitability (Madamba et al., 1999) is consistent with this statement, and changes in dynorphins further supports the hypothesis that alcohol abuse may engage molecular mechanisms of synaptic plasticity in the dorsal striatal sub-region responsible for habit formation.

The incentive-sensitization model of addiction postulates that processing of drug/alcohol cues shifts from ventral to dorsal striatum during the transition from goal-directed (reward driven, “wanting”) to habitual and compulsive drug/alcohol use (Everitt and Robbins, 2005; Vollstadt-Klein et al., 2010). Thus, compulsive alcohol use is under control of the dorsal striatum. The N. accumbens mediates motivational and affective functions while the dorsal striatum encompasses the association and sensorimotor domains (Siciliano et al., 2015). Chronic ethanol exposure causes neuroadaptations in the dorsal striatum that prime for greater control over learning. This shift to striatal dominance over behavior may be critical for development of alcohol use disorders (Depoy et al., 2013). The EOS is prominent in the dorsal striatum. Synthetic and endogenous opioid peptides induced robust long-term depression of excitatory inputs to the dorsal striatum (Atwood et al., 2014). MOR, DOR, and KOR activation produced distinct forms of this depression. KOR-mediated depression was subregion specific while MOR- and DOR-mediated effects were specific for input suggesting the different roles of the three opioid receptors in regulation of specific components of striatal-based behaviors. Several human and animal lines of evidence support this statement regarding alcoholism (Weerts et al., 2011; Nielsen et al., 2012; Ray et al., 2014; Siciliano et al., 2015). Thus, PET imaging of MOR and DOR in alcohol-dependent and control subjects demonstrated the increase in binding potential of the MOR-selective ligand carfentanil in alcoholics providing evidence of a prominent role of the MOR in alcohol dependence (Weerts et al., 2011). Similarly with the molecular differences between alcoholics and controls identified in the present study, the increase in binding potential may (i) represent a predisposing risk factor for alcohol dependence; or (ii) be result of long-term drinking and alcohol dependence. The binding potential of DOR-selective ligand methylnaltrindole did not differ between the groups however, in caudate it positively correlated with recent alcohol drinking in alcohol-dependent subjects. Thus, the DOR activity may be affected by recent alcohol drinking history.

The putamen and the precommissural dorsolateral caudate receive input from the sensorimotor cortex and mediate habitual drug-taking behaviors that develop after chronic administration. Understanding the dopaminergic inputs to these striatal regions may shed light on a role of dopaminergic signaling in alcoholism. Indeed, voluntary ethanol intake in macaques was found to induce the KOR supersensitivity and regionally specific dopaminergic adaptations in the striatum (Siciliano et al., 2015). Both dopaminergic neurotransmission and KOR sensitivity were dysregulated in the N. accumbens and dorsolateral caudate in ethanol drinking animals. Dopamine release and uptake were increased in the N. accumbens while decreased in the dorsolateral caudate. In drinking animals both areas developed KOR sensitivity. The development of KOR sensitivity in the dorsolateral caudate in macaques (Siciliano et al., 2015) and increase in MOR binding potential in dorsal striatum in human alcoholics (Weerts et al., 2011) may develop due to (i) the elevation in a number of KOR and MOR binding sites; or (ii) the decrease in synthesis of their endogenous ligands dynorphins and enkephalins. Our data on the decrease in the levels of these peptides in the dorsal striatal areas in alcoholics corroborates the former hypothesis.

We previously demonstrated that the *PDYN* promoter SNP rs1997794 associated with alcohol dependence may form non-canonical AP-1 binding site and influence gene expression in human brain (Taqi et al., 2011). In present study, the combined CC and CT genotypes but not the TT genotype showed downregulation of both *PDYN* expression and levels of PDYN derived LER opioid peptide in the putamen of alcoholics, while no significant effect of this SNP in the caudate nucleus was evident. Thus, the impact of rs1997794 on *PDYN* transcription may be relevant for brain-area specific adaptive responses of this gene to alcohol. One may speculate that the upregulation of delta-FOSB, a constituent of AP-1 that was proposed as a general mechanism of addiction (Nestler et al., 2001) may contribute to *PDYN* regulation if this transcription factor activates *PDYN* expression by binding to the T allele of *PDYN* promoter SNP (rs1997794). However, this is not apparently the case because delta-FOSB (i) has not been identified as a subunit of AP-1 in the human brain where it is formed by the JUND/FOSB heterodimer (Taqi et al., 2011), (ii) is expressed at negligible levels compared to FOSB in the human brain, and (iii) is not elevated in the brain of alcoholics (Watanabe et al., 2009).

We previously reported that in the human anterior cingulate cortex correlations between *PDYN* mRNA vs. Dyn A and *PDYN* mRNA vs. Dyn B levels were high and significant (*R* = 0.70–0.85; *p* < 0.001) whereas those between the respective mRNA vs. LER and MEAP were substantially lower (Table 3 in Watanabe et al., 2015). In the present study these four correlations were relatively strong (*R* = 0.46–0.73; *p* < 0.001) for both the caudate nucleus and putamen in both studied groups of subjects (Supplementary Figure S1). The correlations differed between brain areas suggesting that enzymes that process the precursor protein molecules and convert longer opioid peptides to shorter enkephalins play a role in regulation of tissue peptide levels (Watanabe et al., 2015). Another factor that may influence the correlation strength is anatomical structure; the PDYN and PENK molecules may be processed to dynorphins and enkephalins either at dendritic location in the expressing neurons, or in their terminals projecting to another areas, and this could result in an imbalance between the mRNA and peptide levels. Consistently, the discrepancy between changes in the mRNA and peptide levels in the caudate and putamen of alcoholics may be due to the alterations in (i) activity of enzymes metabolizing the opioid peptides, and/or (ii) features of intracellular trafficking of the protein precursor molecules from the analyzed areas in the pathological human brain.

As the limitations, the identified associations may be only applicable (a) to males because no female subjects were analyzed, and (b) to 83% of analyzed individuals because 10 out of 60 subjects were excluded from analysis. These ten individuals were identified as overly influential by applying the general statistical approach (Faraway, 2002). They may represent distinct subject subgroups in a heterogeneous human population, or may have clinical and demographic characteristics not described in their medical history or tissue characteristics altered during tissue processing. As a general practice, such subjects and also clinical, biological and technical outliers are excluded from analysis in molecular human brain studies (Ernst et al., 2009; Shulha et al., 2012). Consistently, a design of molecular case-control studies based on analysis of subgroups of subjects but not mean group effects due to heterogeneity of human population and etiological heterogeneity of a disease was proven to be successful in characterization of neuropsychiatric disorders (Sebat et al., 2007; Walsh et al., 2008; Ernst et al., 2009; Shulha et al., 2012). Another limitation is the size of the sample and genetic origin of the individuals analyzed; two group consisted of 24 and 26 subjects, respectively, altogether 50 subjects, all European descent were included in the final statistical analysis. As always with human post-mortem molecular investigations, the present study requires a replication with unrelated set of specimens with characterized genetic background and identified ethnicity that is practically challenging. Therefore, this work may be considered as a pilot study.

Chronic activation of distinct cellular mechanisms by different addictive substances may induce some shared molecular adaptations - the common molecular syndrome in the brain regions mediating the lasting nature of the addictive state (Robison and Nestler, 2011). Downregulation of the dynorphin system in the dorsal striatum found in cocaine addicts (Yuferov et al., 2009) and alcoholics (the present study) may be a part of this shared adaptive mechanism.

### Conflict of interest statement

The authors declare that the research was conducted in the absence of any commercial or financial relationships that could be construed as a potential conflict of interest.
