# Supplementary material for: Downregulation of the endogenous opioid peptides in the dorsal striatum of human alcoholics
Source: Front Cell Neurosci. 2015 May 12;9:187. doi: 10.3389/fncel.2015.00187 (PMC4428131; doi:10.3389/fncel.2015.00187)
Supplement: Supplementary file 1 [file DataSheet1.DOCX]

***Supplementary material***

**Downregulation of the endogenous opioid peptides in the dorsal striatum of human alcoholics**

**Daniil Sarkisyan^1,^*^,#^, Muhammad Z. Hussain^2,#^, Hiroyuki Watanabe^1,#^, Olga Kononenko^1,3^, Igor Bazov^1^, Xingwu Zhou^1^,** **Olga Yamskova^4^, Oleg Krishtal^3^, Victor M. Karpyak^5^, Tatiana Yakovleva^1^, Georgy Bakalkin^1^**

^1^Division of Biological Research on Drug Dependence, Department of Pharmaceutical Biosciences, Uppsala University, Uppsala, Sweden

^2^Government degree college, Makhdoom Rasheed, Multan, Pakistan

^3^Department for Cellular Membranology, Bogomoletz Institute of Physiology, Kyiv, Ukraine

^4^Department of Functional Pharmacology, Institute for Neuroscience, Uppsala University, Uppsala, Sweden

^5^Department of Psychiatry and Psychology, Mayo Clinic, Rochester, MN, USA

*** Correspondence:** Daniil Sarkisyan, Division of Biological Research on Drug Dependence, Department of Pharmaceutical Biosciences, Uppsala University, Box 591, Uppsala, 751 24, Sweden.

daniil.sarkisyan@farmbio.uu.se

# Authors contributed equally to this work

**Supplementary Table S1.** Association of *PDYN* promoter SNP rs1997794 variants with alcohol dependence.

| **Genotype** | **Number of subjects** | | **P value*** | |
| --- | --- | --- | --- | --- |
|  | **Controls (n = 26)** | **Alcoholics (n = 24)** | **Genotype test** | **Allele test** |
| TT | 10 | 11 | 0.79 | 0.73 |
| CT | 12 | 11 |  |  |
| CC | 4 | 2 |  |  |

*The Fisher′s exact test was applied to assess significance of associations.

**Supplementary Table S2.** Primer sequences used for real-time PCR amplification.

| **Gene symbol** | **Forward primer 5′→3′** | **Reverse primer 5′→3′** | **Reference** | **Amplicon size (bp)** |
| --- | --- | --- | --- | --- |
| *PENK* | CGG TTC CTG ACA CTT TGC ACT | CAC ATT CCA TTA CGC AAG CC | ([Backman et al.. 2007](#_ENREF_1)) | 151 |
| *PDYN* | CAC CAC AGC GGA CTG CCT GT | AGC AGG GCA GCC TGG CAT TG | * | 111 |

*The primers were designed using Vector NTI advance 10.3.1 software (Invitrogen).

**References**

Backman. C.M.. Shan. L.. Zhang. Y.. Hoffer. B.J.. and Tomac. A.C. (2007). Alterations in prodynorphin. proenkephalin. and GAD67 mRNA levels in the aged human putamen: correlation with Parkinson's disease. *J Neurosci Res* 85**.** 798-804. doi: 10.1002/jnr.21164.

**Figure legend**

**Supplementary Figure S1** Correlations between *PDYN* mRNA and PDYN derived peptides Dyn A **(A)**, Dyn B **(B)** and Leu-enkephalin-Arg (LER) **(C);** and between *PENK* mRNA and PENK derived Met-enkephalin-Arg-Phe (MEAP) **(D)** in the caudate nucleus and putamen of alcoholics and controls.
