# Supplementary figures and images for: Downregulation of the endogenous opioid peptides in the dorsal striatum of human alcoholics
Source: Front Cell Neurosci. 2015 May 12;9:187. doi: 10.3389/fncel.2015.00187 (PMC4428131; doi:10.3389/fncel.2015.00187)

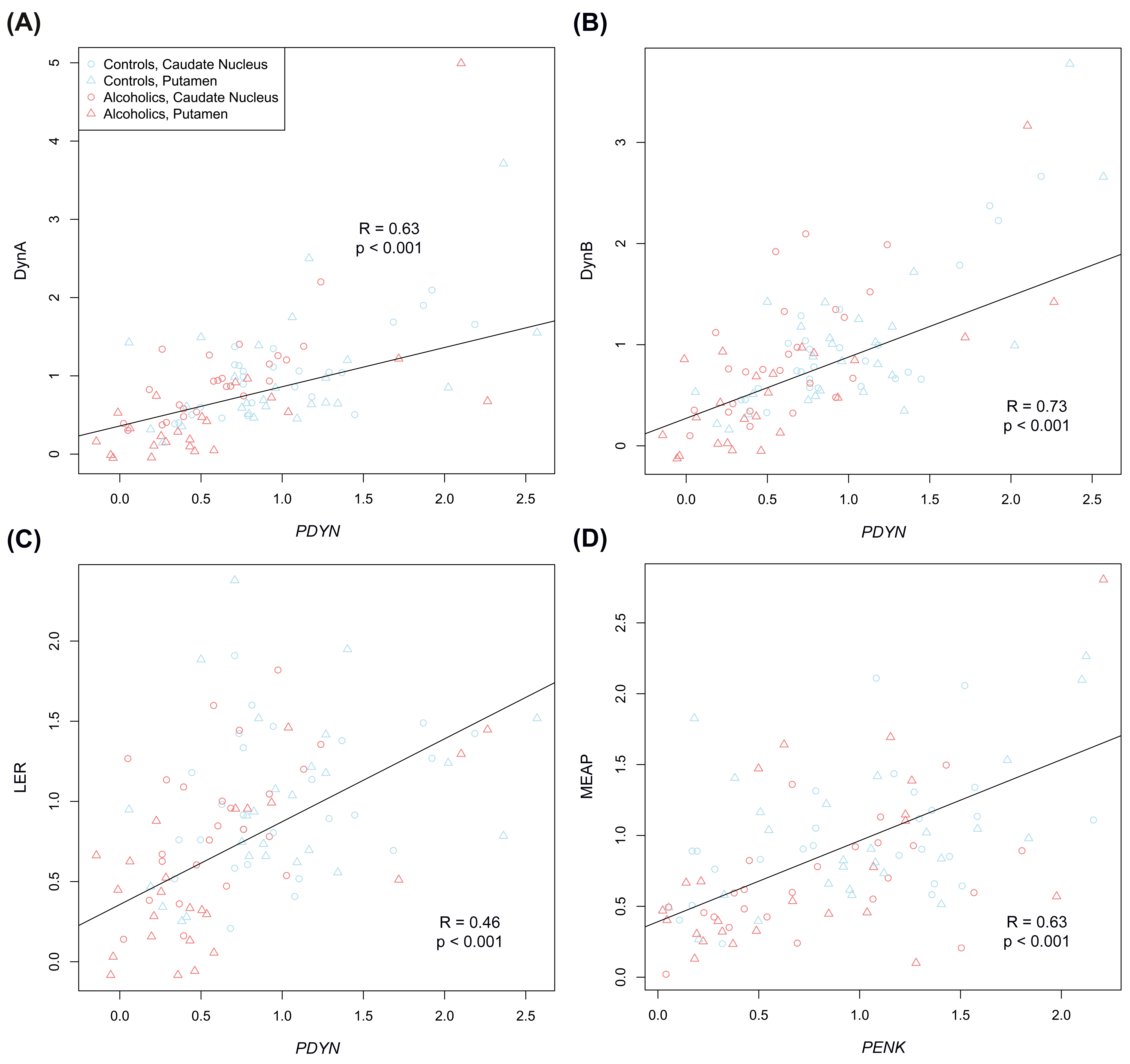

Supplement: Supplementary file 2 [file Image1.TIF]
